# Postprandial effects of a whey protein-based multi-ingredient nutritional drink compared with a normal breakfast on glucose, insulin, and active GLP-1 response among type 2 diabetic subjects: a crossover randomised controlled trial

**DOI:** 10.1017/jns.2021.41

**Published:** 2021-07-12

**Authors:** Pimnapanut Sridonpai, Aree Prachansuwan, Kemika Praengam, Siriporn Tuntipopipat, Wantanee Kriengsinyos

**Affiliations:** Institute of Nutrition, Mahidol University, Nakhon Pathom, Thailand

**Keywords:** Acute glycaemic response, Breakfast meal, Glucagon-like peptide-1, Whey protein, Xylitol, BC, boiled white rice with chicken, BMI, body mass index, GLP-1, glucagon-like peptide-1, iAUC, incremental area under the curve, min, minute, sem, standard error of the mean, T2DM, type 2 diabetes mellitus, WD, whey protein-based multi-ingredient nutritional drink

## Abstract

Postprandial hyperglycaemia is recognised as an important target in type 2 diabetes management. Dietary pattern, meal composition, and amount of food intake are major factors for maintaining postprandial blood glucose levels. The aim of this study was to investigate the effect of consuming a whey protein-based multi-ingredient nutritional drink (WD) on postprandial glycaemic, insulinaemic, and active glucagon-like peptide-1 (GLP-1) responses in comparison to a typical breakfast, which is boiled white rice with chicken (BC) in patients with type 2 diabetes mellitus (T2DM). Fifteen subjects with T2DM participated in a randomised, controlled, cross-over study. Two isocaloric diets with similar nutrient composition were randomly tested with at least 7 d in between. Glucose, insulin, and active GLP-1 were measured by standard methods with blood samples collected with a venous catheter for 240 min during a kinetic test. The incremental area under the curve (iAUC_0–240 min_) for plasma glucose was significantly lower after the consumption of WD (WD: 3551 ± 546; BC: 9610 ± 848 mg min/dl; *P* < 0⋅01), while insulinaemic response tended to be lesser (iAUC_0–240 min_) than those of BC. In addition, higher iAUC_0–240 min_ for active GLP-1 was obtained with WD diet (WD: 2230 ± 441; BC: 925 ± 183 pM min/ml; *P* < 0⋅01). This study showed that WD can be used to replace a regular breakfast for improving postprandial glucose response and active GLP-1 levels in people with T2DM. Further studies are required to elucidate the clinical efficacy of WD on long-term glycaemic control in people with T2DM.

## Introduction

Type 2 diabetes mellitus (T2DM) is a global public health problem worldwide, which is characterised by chronic hyperglycaemia. Postprandial hyperglycaemia might be an independent risk factor for both microvascular and macrovascular complications of T2DM and is the major determinant for the level of glycated haemoglobin^(1)^. Persistent lowering of postprandial plasma glucose can improve glycaemic control and prevent serious life-threatening complications^(2)^. Postprandial glycaemic response is provoked by the diet composition, mainly the type and amount of food.

It has been well established that slowly digestible carbohydrates can prolong the releasing of glucose into the bloodstream and attenuating the postprandial hyperglycaemia, consequently using as main carbohydrate sources in diabetic-specific enteral formulas or drinks^(3,4)^. Consumption of the high-slowly digestible starch (SDS) for breakfast showed a positive effect on lower postprandial glycaemic and insulinaemic responses, in comparison to consuming the low-SDS products^(5)^. Likewise, sugar alcohols are also recognised as low-glycaemic index (GI) sweeteners commonly used to substitute sucrose in food products for increased variety in food choices and the quality of life for people with diabetes^(6)^. Sugar alcohols or foods sweetened with sugar alcohols ingestion provided a favourable effect on acute glycaemic and insulinaemic responses in healthy subjects^(7)^ and T2DM patients^(8)^. However, protein and/or amino acid consumption is another influential factor on postprandial glycaemic control in both nondiabetic and diabetic subjects^(9)^.

Remarkably, previous reports revealed that the consumption of whey protein alone (40–60 g) or co-ingested with carbohydrates together can reduce glycaemic response^(10–12)^. It has been proposed that whey protein-based breakfast should be considered as a management strategy in patients with T2DM^(13–15)^ due to stimulating insulin secretion and augmenting incretin effect through glucagon-like peptide 1 (GLP-1) secretion^(13,16,17)^. Co-ingestion of whey protein (18 or 20 g) with a high-GI meal increased the insulinaemic response to that meal, compared with a meal without whey protein in healthy subjects^(18,19)^. In diabetic patients, Frid *et al.*^(16)^ suggested that the co-ingestion of whey proteins (5⋅3 g) and a high-GI breakfast did not differ from the reference breakfast in terms of glycaemic and insulinaemic responses. Nevertheless, 15-g whey protein co-ingested with breakfast containing whole-grain cereal and whole milk significantly diminished the area under the curve (AUC) for plasma glucose by 13 % along with the elevation in plasma insulin after breakfast when compared with whole-grain cereal and whole milk without whey protein^(20)^. It seemed that co-ingestion of a low-GI carbohydrate and whey protein-containing meal might synergistically improve glycaemic and insulinaemic responses in people with T2DM.

To promote better glycaemic responses, low-GI carbohydrates and whey protein can be used as the main ingredients in the meal replacement food products^(21)^. Using low-glycaemic responses, liquid meal replacement might be an alternative dietary approach to improve postprandial hyperglycaemia for T2DM management^(22)^. Previous intervention studies in Caucasians with T2DM have been demonstrated that isocaloric breakfast replacement with a low-GI liquid formula provided better postprandial glycaemic control, insulin sensitivity, and plasma GLP-1 responses when compared with semi-solid oatmeal^(23,24)^. However, a comparison of liquid meal replacement to Asian whole food breakfast or rice-based breakfast on postprandial glucose, insulin, and active GLP-1 responses has not been explored.

The present study was aimed to compare the impact of a whey protein-based multi-ingredient nutritional drink (WD), mainly consisting of whey protein, sugar alcohols, and slowly digestible carbohydrates, on postprandial glycaemic, insulinaemic, and plasma active GLP-1 responses in comparison to an Asian common breakfast, which is boiled white rice with chicken (BC) in Thai patients with T2DM.

## Materials and methods

### Study design

This was a randomised, open-label, cross-over clinical trial. Subjects were randomly assigned to two different test meals with 1-week washout period using a computer-generated list of random numbers (Microsoft Excel, Washington, DC, USA). This study was conducted according to the guidelines laid down in the Declaration of Helsinki, and all procedures involving human subjects were approved by the Central Institutional Review Board of Mahidol University No. MU-CIRB2019/156.0606. Written informed consent was obtained from all subjects before inclusion in the study. The study was registered at clinicaltrials.in.th as TCTR20190917004.

### Study participants

Fifteen Thai subjects were recruited via poster advertisements at the Salaya Campus of Mahidol University, Thailand. Eligibility criteria were a diagnosis of T2DM according to self-report, aged 20–60 years, body mass index (BMI) 18⋅5–30 kg/m^2^, use of oral glucose-lowering agents except any GLP-1 analogues, and no current insulin use. The exclusion criteria included renal or hepatic disease, thyroid disorder, pregnant or lactating women, and unstable body weight, which were defined as 2 kg change within 2 months. Subjects who were taking oral steroids in the past 3 months, antibiotic medication in the past 3 weeks, and dietary supplements, were excluded. All subjects were interviewed by telephone for general information and medication uses.

### Study protocol

At the first visit, body weight, height, BMI, and body composition were assessed individually. Subjects fasted overnight for 10 h before blood collection. Fasting plasma glucose, serum insulin, and Homeostasis Model Assessment-Insulin Resistance (HOMA-IR) were presented for the general characteristics.

**Fig. 1.**
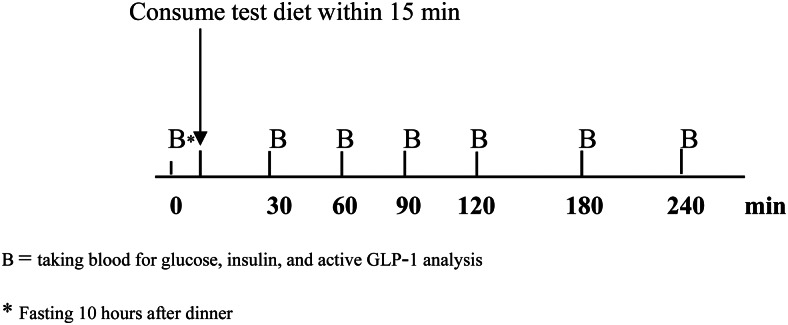
Study protocol.

In each study visit, subjects were asked to avoid strenuous exercise and refrain from smoking and drinking alcoholic beverages for 24 h before the experiment day. They were instructed to standardise their evening meal prior to the study visits. After a 10-h overnight fast, they were asked to attend the clinical research unit at 08.00 h. Their capillary blood glucose was measured by a glucometer (Accu-Chek Active, Roche Diagnostics, Indianapolis, IN, USA) before starting the experiment. If the value was >180 or <70 mg/dl, the experiment would be precluded. The study protocol was carried out as shown in Fig. 1. Blood samples were drawn through an indwelling cannula in the antecubital fossa to obtain baseline measurements. After that, subjects took their usual medications (if any) with 150 ml drinking water and then consumed the test diet within 15 min. Subsequent blood samples (5 ml) were drawn at 30, 60, 90, 120, 180, and 240 min after the breakfast test. Plasma glucose, serum insulin, and plasma active GLP-1 concentrations were analysed and plotted to capture time-course changes from baseline.

### Dietary interventions

Subjects were given either WD or BC. The two test diets were isocaloric meals with the similar amount of carbohydrate, protein, fat, and dietary fibre. Composition of the test diets is presented in Table 1. WD was prepared using 100 g of a vanilla-flavored instant beverage (GLUCOMEAL^™^) dissolved in 400 ml water. A typical Thai breakfast named BC was cooked based on the standard recipe in the metabolic kitchen at the Institute of Nutrition, Mahidol University. The serving size of each test diet and their nutritive values are given in Table 2.

**Table 1.** Composition of test diets

Boiled white rice with chicken; BC		Whey protein-based multi-ingredient nutritional drink; WD	
Raw white rice polished	46⋅73 %	WPC (80 % whey protein)	22⋅00 %
Raw breast chicken without skin	29⋅91 %	MCT oil	15⋅00 %
Palm oil	8⋅10 %	Xylitol	23⋅89 %
Soaked mushroom dried (shiitake)	3⋅12 %	Maltitol	14⋅41 %
Soybean sauce (formula 1)	3⋅12 %	Isomaltulose	11⋅00 %
Garlic	3⋅12 %	Inulin	5⋅00 %
Raw celery	1⋅87 %	Xanthan gum	0⋅78 %
Seasoning sauce (grade 1)	1⋅87 %	Vitamin/mineral Premix	0⋅50 %
Coriander root	1⋅25 %	Inositol	0⋅38 %
Dried fermented cabbage (Tang-chai)	0⋅62 %	Taurine	0⋅04 %
White pepper, powder	0⋅31 %	Mixture of probiotics	0⋅06 %
		Other ingredients: Maltodextrin, INS415, INS339(i), cream flavour, vanilla flavour	6⋅94 %

WPC, whey protein concentrate; MCT, medium-chain triglyceride.

**Table 2.** Nutritive values per serving of test diets^a^

Parameters	Boiled white rice with chicken; BC	Whey protein-based multi-ingredient nutritional drink; WD
Serving size, g	484	500
Energy, kcal	444	442
Carbohydrate, g	64	66
Dietary fibre, g	1⋅40	1⋅28
Protein, g	18	17
Fat, g	13	12

a Nutrient composition for WD was obtained from Mega Lifesciences Pty. Ltd. (Thailand), whereas nutrient composition for BC was obtained from the chemical analysis data analysed by the ALS laboratory Group (Thailand) Co., Ltd.

### Blood analysis

Whole blood samples were collected into sodium fluoride containing tubes and clotted blood tubes. The plasma/serum was separated within 15 min by centrifugation at 2000 ***g*** for 10 min. Plasma glucose was measured by the enzymatic method (Cobas 6000 (c702) analyser, Roche Diagnostic GmbH, Mannheim, Germany). The intra-assay and inter-assay coefficient of variations (CVs) for plasma glucose were 0⋅03 and 1⋅10 %, respectively. Serum insulin was measured using Electrochemiluminescence Immunoassay (ECLIA) (Cobas 6000 (e602) analyser, Roche Diagnostic, GmbH, Mannheim, Germany). The intra-assay and inter-assay CVs for serum insulin were 0⋅03 and 2⋅21 %, respectively.

Another sample of whole blood was collected into an EDTA plasma tube for active GLP-1 analysis. The dipeptidyl peptidase IV inhibitor (EMD Millipore Corporation, USA) [10 μl/ml blood] was immediately added, mixed (<30 s), and centrifuged (1000 ***g*** for 15 min at 4 °C). Plasma active GLP-1 was measured by the sandwich ELISA kit (EGLP-35K, EMD Millipore Corporation, Billerica, MA, USA) according to the manufacturer's protocol with a sensitivity of 0⋅14 pM. The concentration of active GLP-1 was calculated from a standard curve. The intra-assay and inter-assay CVs for plasma active GLP-1 were 7 and 9⋅8 %, respectively.

### Statistical analysis

#### Sample size

A sample size of 15 subjects was estimated based on the mean (±standard deviation) difference in postprandial glucose AUC levels after consumption of the diabetes-specific formula, which is 3412 ± 2608 mg min/dl in Caucasian T2DM patients, with a power of 95 %, a significant level of 0⋅05, and dropout of 30 %^(24)^.

#### Data analysis

Statistical analysis was performed using SPSS software version 18.0 (IBM Corp., Armonk, NY, USA). Data were expressed as mean ± standard error of the mean (sem). Two-tailed *P* <0⋅05 was considered significant. Distribution of the data was analysed according to the Shapiro–Wilk test. Incremental area under the curves for plasma glucose, insulin, and active GLP-1, from 0 to 240 min after diet test intake (iAUC_0–240 min_), was calculated geometrically using the trapezoidal rule via Prism version 5.01 (GraphPad, San Diego, CA, USA). Repeated-measures ANOVA test was used to evaluate the postprandial plasma glucose, insulin, and active GLP-1, testing for time × treatment interactions and the effect of time and test meals separately. Statistical paired *t*-tests were used to analyse the mean difference of iAUC_0–240 min_ for glucose, insulin, and active GLP-1 after consumption of two test meals.

## Results

### General characteristics of subjects

Fifteen Thai subjects (nine males and six females) were completed the study and general characteristics are shown in Table 3. Subjects had a mean age of 50⋅1 years (sem, 1⋅6 years; range, 40–57 years), had controlled T2DM with the mean duration of 4⋅14 years (sem, 0⋅49 years; range 2–8 years), and BMI of 26⋅6 kg/m^2^ (sem, 1⋅1 kg/m^2^; range, 19⋅7–32⋅8 kg/m^2^). The average of fasting plasma glucose and HOMA-IR was 133⋅4 (sem, 6⋅7) mg/dl and 5⋅58 (sem, 0⋅83), respectively. No complaints or no clinical difference in signs or symptoms were observed during the study. All participants appeared to tolerate well both test diets.

**Table 3.** General characteristics of T2DM subjects participated in the study

Parameters	Values
Gender (male/female), *n* (%)	9 (60⋅0)/6 (40⋅0)
Age, years	50⋅1 ± 1⋅6
BMI, kg/m^2^	26⋅6 ± 1⋅1
Fasting plasma glucose, mg/dl	133⋅4 ± 6⋅7
HOMA-IR	5⋅58 ± 0⋅83
Body composition	
Lean body mass, kg	45⋅5 ± 2⋅8
Body fat, %	32⋅7 ± 2⋅3
Visceral fat area, cm^2^	123⋅8 ± 8⋅0
Glucose-lowering medication	
Only metformin, *n* (%)	9 (60⋅0)
Metformin and sulfonylurea, *n* (%)	2 (13⋅3)
Metformin and thiazolidinediones, *n* (%)	2 (13⋅3)
Metformin, sulfonylurea, and thiazolidinediones, *n* (%)	1 (6⋅7)
Metformin, sulfonylurea, SGLT-2 inhibitor, *n* (%)	1 (6⋅7)

Values are number (%) or mean ± sem; *n* 15.

HOMA-IR, Homeostasis Model Assessment of Insulin Resistance.

With regard to oral antihyperglycaemic medications, 9 patients (60⋅0 %) were using metformin only, 2 patients (13⋅3 %) were using metformin plus sulfonylurea, 2 patients (13⋅3 %) were using metformin plus thiazolidinedione, 1 patient (6⋅7 %) was using metformin, sulfonylurea, and thiazolidinedione, and 1 patient (6⋅7 %) was using metformin, sulfonylurea, and SGLT-2 inhibitor.

### Postprandial plasma glucose response

**Fig. 2.**
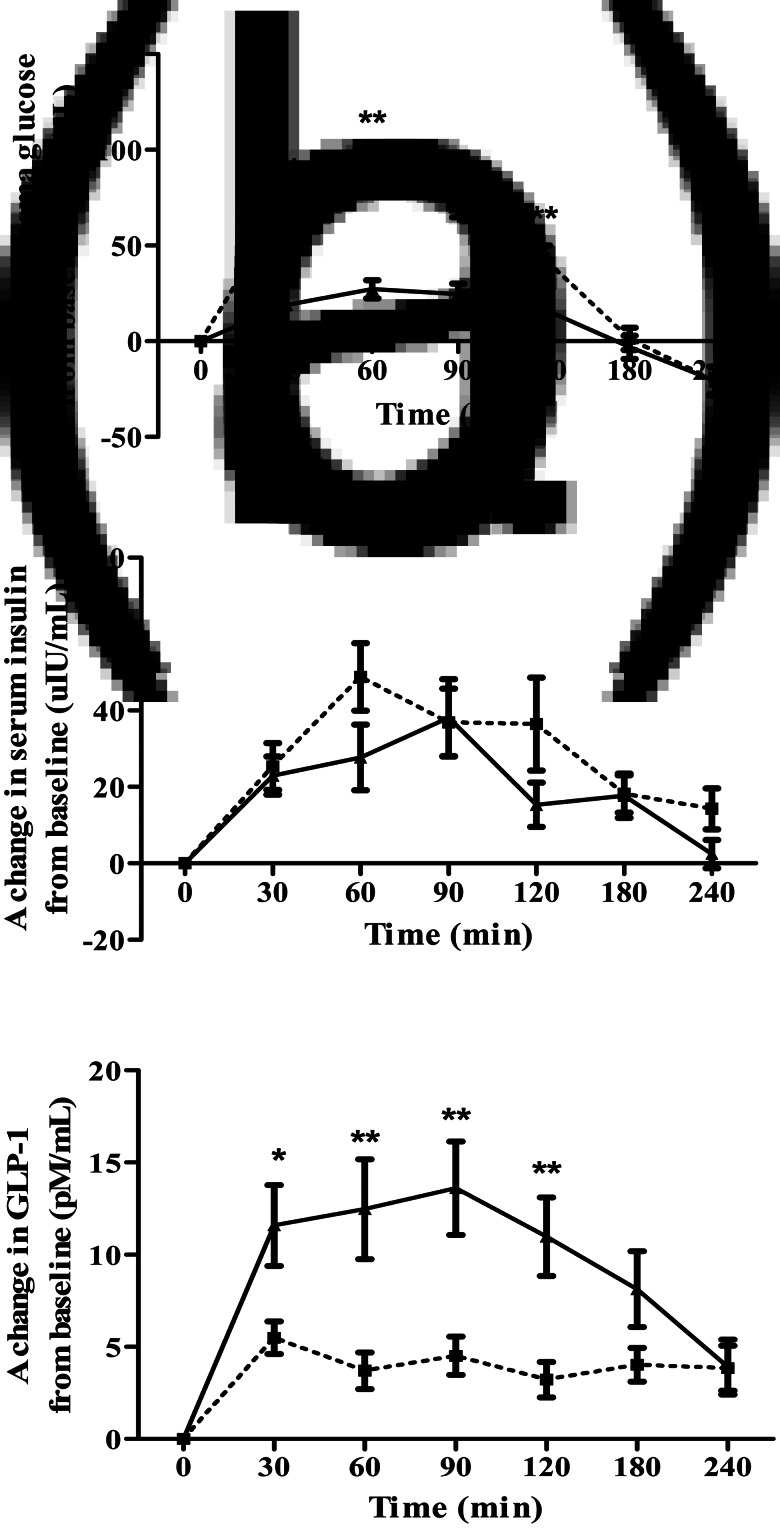
Acute response effects on plasma glucose (a), serum insulin (b), and plasma GLP-1 (c) above the baseline after consuming either BC (□) or WD (▴). Values are mean ± sem; *n* 15. Mean values are significantly different from each other: *P < 0⋅05, **P < 0⋅001, which was determined by repeated-measures ANOVA with Bonferroni's correction for *post hoc* comparisons between test diets.

A change of postprandial glucose responses and iAUC_0–240 min_ for glucose are illustrated in Fig. 2(a) and Table 4, respectively. The mean values of plasma glucose at baseline and after consumed test meal are shown in Supplementary Table S1 of Supplementary material. Postprandial incremental glucose levels after the consumption of the BC meal were significantly higher than those after the WD meal at 30, 60, 90, and 120 min. Noticeably, plasma glucose had increased moderately and reached a plateau during 30–120 min after consumption of the WD meal. A 63 % reduction in the area under the curve (iAUC_0–240 min_) for glucose was observed after the WD consumption compared with BC (*P* < 0⋅001).

**Table 4.** Postprandial plasma incremental area under the curve (iAUC) for plasma glucose, serum insulin, and plasma active GLP-1 in response to the different breakfasts in T2DM subjects

Parameters	Boiled white rice with chicken, BC	Whey protein-based multi-ingredient nutritional drink, WD
Glucose iAUC (mg min/dl)	9610 ± 848	3551 ± 546^a^
Insulin iAUC (μIU min/ml)	6582 ± 1051	4802 ± 976
Active GLP-1 iAUC (pM min/ml)	925 ± 183	2230 ± 441^a^

Values are mean ± sem; *n* 15.

iAUC, incremental area under the curve after consumed test diets (0–240 min) calculated using Prism version 5⋅01 (GraphPad, San Diego, CA, USA). Mean values are significantly different from each other:

a *P* < 0⋅001 which was determined by the paired sample *t*-test.

### Postprandial serum insulin response

There was no significant distinction between the WD and BC meal in postprandial insulin responses between 0 and 240 min (see Fig. 2(b)). Incremental area under the insulin curve over 4 h was approximately 27 % lower in WD than that of BC but did not show a statistically significant difference as reported in Table 4. The mean values of serum insulin at baseline and after consumed test meal are given in Supplementary Table S2 of Supplementary material.

### Postprandial plasma active GLP-1 response

Postprandial plasma active GLP-1 levels after consumption of the WD meal were significantly higher than those after the BC meal at 30, 60, 90, and 120 min as shown in Fig. 2(c). Incremental area under the plasma active GLP-1 curve during the entire study period elicited significantly 141 % higher for WD diet than that of BC (*P* < 0⋅001) as described in Table 4. The mean values of plasma active GLP-1 at baseline and after consumed test meal are provided in Supplementary Table S3 of Supplementary material.

## Discussion

The present study shows that the ingestion of a whey protein-based multi-ingredient nutritional drink (WD) improved postprandial glycaemic and active GLP-1 responses when compared with regular breakfast in volunteers with T2DM, whereas insulin changes indicated no significant difference between those two test diets. Consumption of WD significantly produced a lesser increment of postprandial plasma glucose and stimulated a greater GLP-1 secretion than those of an Asian common breakfast. These findings emphasised the impact of a liquid meal replacement on postprandial glucose excursions, consequently providing benefits for people with T2DM.

This observation may be explained by the difference in the type of proteins and carbohydrates. Even though the two breakfast diets contain the similar content of energy and macronutrients (carbohydrate, protein, fat, and dietary fibre), both of them were obtained from the different ingredients. WD was prepared using GLUCOMEAL^™^, which consisted of whey protein concentrate, and several kinds of carbohydrates including xylitol, maltitol, isomaltulose, inulin, xanthan gum, as well as maltodextrin, whereas chicken and white rice were the main composition for BC. It might be affirmed that the different dietary components have significant impacts on blood glucose regulation.

It has been suggested that consuming whey protein is considered as a potential application for the treatment of obesity and T2DM^(15)^. A possible mechanism has been proposed which indicates that whey protein slows down gastric emptying, and stimulates GLP-1 secretion^(25)^. Furthermore, whey protein is an abundant source of amino acids and bioactive peptides which can directly augment the insulin secretory response to meal ingestion, thereby attenuating postprandial plasma glucose^(26)^. Our results found that consuming a 17-g whey protein-containing drink provided higher GLP-1 secretion (+141 %), lower insulin levels (−27 %), and better glycaemic responses (−63 %) than those of chicken/rice protein-based meal. Stimulation of GLP-1 secretion might be attributed from dairy proteins containing higher concentrations of branch-chain amino acids (BCAAs) and unique bioactive peptides than those of other animal proteins, accordingly providing the beneficial effects on glucoregulatory outcomes^(27)^. It was affirmed that the individual BCAAs, leucine and isoleucine, and a mixture of essential amino acids can efficiently induce GLP-1 release from the human intestinal cell line^(28,29)^. This observation may be involved with the mitogen-activated protein kinases (MAPKs) in regulating the endogenous GLP-1 secretion from the L cells of colonic mucosa^(29)^.

Co-ingestion of dairy protein with a meal has strongly augmented postprandial insulin concentration and attenuated the postprandial rise in glucose levels in T2DM patients^(30)^. A 64 % increase in insulinaemia was observed after consumption of 18 g whey proteins served with 25 g of glucose solution when compared with the reference drink without whey protein among healthy volunteers^(19)^. Among T2DM men, King *et al.*^(20)^ supported that a dose (15 g) of whey protein co-ingested with breakfast meal stimulates insulin release (+20 %) and attenuates postprandial glycaemia AUC (−13 %) compared with the breakfast meal, consisting of 60 g of whole-grain cereal and 250 ml of whole milk. These obvious results suggested that types of carbohydrate co-ingested with whey protein may mask the effects on insulin response even if whey protein is reported as an efficient insulin secretagogue in people with T2DM^(31)^.

In our study, a mixture of carbohydrates, including xylitol, maltitol, isomaltulose, inulin, xanthan gum, as well as maltodextrin, was used for WD, while BC mainly comprised of cooked white rice (Thai Hom Mali fragrant rice), which has been reported to have a high GI (96 %) among healthy population^(32)^. Noticeably, types of carbohydrate can have an effect on postprandial glycaemia beyond the adequate quantity^(33)^. A previous meta-analysis study revealed that consumption of the high-SDS for breakfast was strongly associated with lower postprandial glycaemic and insulinaemic responses, compared with consuming the low-SDS products^(5)^. In addition, non-digestible oligosaccharides, resistant starch, and sugar alcohols also produced lesser postprandial glucose levels in patients with T2DM^(34)^. However, the small amount of dietary fibre in our test meals was unlikely the cause of the differential glucose responses obtained in this study, as reported by the study of Nuttall^(35)^. Therefore, it seemed that the different acute responses on plasma glucose and insulin in this study might be attributed to the carbohydrate mixture of SDS, inulin, and sugar alcohols in WD.

With the magnitude of carbohydrate mixture, sugar alcohols are the main proportion of the carbohydrates in the WD meal particularly xylitol. Xylitol has been considered as a low-GI carbohydrate, which can stimulate very little in acute glycaemic and insulinaemic responses^(36)^. Wölnerhanssen *et al.*^(7)^ have also shown that consumption of a 50-g xylitol dissolved in 300 ml of water had a minimal effect on insulin secretion among obese subjects, but the predominant effect on GLP-1 stimulation was observed in both lean and obese subjects. Hence, these effects of sugar alcohols might be an explanation why WD consumption had positive effects on postprandial glucose and active GLP-1 levels but not causing the difference in insulinaemic responses between the meals. Even though xylitol seemed to provide beneficial effects on glycaemic responses among T2DM individuals, its effects on gastrointestinal disturbance in long-term usage needs to be considered.

Our findings were in agreement with Devitt *et al.*^(24)^ who showed that postprandial plasma glucose-positive AUC was significantly reduced by 38 % after consuming glycaemia-targeted specialized-nutrition (GTSN), which was Glucerna Triple Care™, compared with oatmeal as a common breakfast of T2DM patients. Moreover, the GTSN ingestion significantly caused a 280 % higher AUC of postprandial GLP-1 than those of the oatmeal group^(24)^. Consistently, postprandial glycaemic response was attenuated in diabetic patients who consumed either Glucerna™ or Ultra Glucose Control™ compared with oatmeal (*P* < 0⋅001), resulting in the increased GLP-1 secretion without changing for serum insulin levels^(23)^. Nevertheless, the breakfast meals used in both previous studies matched only in energy but not in the amount of carbohydrates, proteins, and fats.

This study has some strengths. First, the cross-over designed study was conducted within the same subjects; therefore, it is likely that the medications have equally affected the study outcomes. Second, two breakfast meals were prepared with isocaloric, iso-macronutrients (carbohydrates, proteins, and fats), and was of similar serving size. However, this study still has several limitations. In our study, the food texture of test meals is different (liquid *v.* semi-solid), so it might influence gastric emptying and gut hormone secretion, leading to different responses of glycaemic control^(37)^. Even though we prepared a similar weight of serving size, the rate of gastric emptying should be further studied. The study design itself could not suggest any long-term improvement in glycaemic parameters. To prove the long-term effects of replacing a common breakfast for a WD drink on glycaemic control and other relevant outcomes for T2DM control such as body weight, a chronic supplementation randomised controlled trial should be performed.

## Conclusion

The present findings indicated that WD provided a better postprandial glvcaemic response and active GLP-1 levels without elevating the insulinaemic excursions compared with ordinary breakfast in patients with T2DM. These could be implied that the nutrition formula containing a mixture of slowly digested carbohydrates and whey protein might be shown to exert beneficial glycaemic management when compared with normal breakfast when used as a meal replacement.

## Supplementary material

For supplementary material accompanying this paper visit http://dx.doi.org/10.1017/jns.2021.41. click here to view supplementary material

## References

[1] Ceriello A (2005) Postprandial hyperglycemia and diabetes complications. Diabetes 54, 1. 15616004 10.2337/diabetes.54.1.1

[2] Blevins T (2011) Control of postprandial glucose levels with insulin in type 2 diabetes. Postgrad Med 123, 135–147. 10.3810/pgm.2011.07.2313 21680998

[3] Vanschoonbeek K, Lansink M, van Laere KMJ, (2009) Slowly digestible carbohydrate sources can be used to attenuate the postprandial glycemic response to the ingestion of diabetes-specific enteral formulas. The Diabetes Educator 35, 631–640. 19448045 10.1177/0145721709335466

[4] Gourineni V, Stewart ML, Skorge R, (2019) Glycemic index of slowly digestible carbohydrate alone and in powdered drink-mix. Nutrients 11, 1228. 10.3390/nu11061228 PMC6627922 31146493

[5] Vinoy S, Meynier A, Goux A, (2017) The effect of a breakfast rich in slowly digestible starch on glucose metabolism: a statistical meta-analysis of randomized controlled trials. Nutrients 9, 318. 10.3390/nu9040318 PMC5409657 28333086

[6] Wolever TM, Piekarz A, Hollands M, (2002) Sugar alcohols and diabetes: a review. Can J Diabetes 26, 356–362.

[7] Wölnerhanssen BK, Cajacob L, Keller N, (2016) Gut hormone secretion, gastric emptying, and glycemic responses to erythritol and xylitol in lean and obese subjects. Am J Physiol Endocrinol Metab 310, E1053–E1061. 27117004 10.1152/ajpendo.00037.2016

[8] Argiana V, Kanellos PΤ, Eleftheriadou I, (2020) Low-glycemic-index/load desserts decrease glycemic and insulinemic response in patients with type 2 diabetes mellitus. Nutrients 12, 2153. 10.3390/nu12072153 PMC7400801 32698325

[9] Russell WR, Baka A, Björck I, (2016) Impact of diet composition on blood glucose regulation. Crit Rev Food Sci Nutr 56, 541–590. 24219323 10.1080/10408398.2013.792772

[10] Anderson GH, Tecimer SN, Shah D, (2004) Protein source, quantity, and time of consumption determine the effect of proteins on short-term food intake in young Men. J Nutr 134, 3011–3015. 15514267 10.1093/jn/134.11.3011

[11] Hall WL, Millward DJ, Long SJ, (2003) Casein and whey exert different effects on plasma amino acid profiles, gastrointestinal hormone secretion and appetite. Br J Nutr 89, 239–248. 12575908 10.1079/BJN2002760

[12] Nilsson M, Stenberg M, Frid AH, (2004) Glycemia and insulinemia in healthy subjects after lactose-equivalent meals of milk and other food proteins: the role of plasma amino acids and incretins. Am J Clin Nutr 80, 1246–1253. 15531672 10.1093/ajcn/80.5.1246

[13] Ma J, Stevens JE, Cukier K, (2009) Effects of a protein preload on gastric emptying, glycemia, and gut hormones after a carbohydrate meal in diet-controlled type 2 diabetes. Diabetes Care 32, 1600–1602. 19542012 10.2337/dc09-0723 PMC2732158

[14] Mortensen LS, Holmer-Jensen J, Hartvigsen ML, (2012) Effects of different fractions of whey protein on postprandial lipid and hormone responses in type 2 diabetes. Eur J Clin Nutr 66, 799–805. 22588635 10.1038/ejcn.2012.48

[15] Jakubowicz D, Wainstein J, Landau Z, (2017) High-energy breakfast based on whey protein reduces body weight, postprandial glycemia and HbA_1C_ in type 2 diabetes. J Nutr Biochem 49, 1–7. 28863364 10.1016/j.jnutbio.2017.07.005

[16] Frid AH, Nilsson M, Holst JJ, (2005) Effect of whey on blood glucose and insulin responses to composite breakfast and lunch meals in type 2 diabetic subjects. Am J Clin Nutr 82, 69–75. 16002802 10.1093/ajcn.82.1.69

[17] Salehi A, Gunnerud U, Muhammed SJ, (2012) The insulinogenic effect of whey protein is partially mediated by a direct effect of amino acids and GIP on β-cells. Nutr Metab 9, 48. 10.1186/1743-7075-9-48 PMC3471010 22647249

[18] Allerton DM, Campbell MD, Gonzalez JT, (2016) Co-ingestion of whey protein with a carbohydrate-rich breakfast does not affect glycemia, insulinemia or subjective appetite following a subsequent meal in healthy males. Nutrients 8, 116. 26927166 10.3390/nu8030116 PMC4808846

[19] Gunnerud UJ, Östman EM & Björck IM (2013) Effects of whey proteins on glycaemia and insulinaemia to an oral glucose load in healthy adults: a dose–response study. Eur J Clin Nutr 67, 749–753. 23632747 10.1038/ejcn.2013.88

[20] King DG, Walker M, Campbell MD, (2018) A small dose of whey protein co-ingested with mixed-macronutrient breakfast and lunch meals improves postprandial glycemia and suppresses appetite in men with type 2 diabetes: a randomized controlled trial. Am J Clin Nutr 107, 550–557. 29635505 10.1093/ajcn/nqy019

[21] Manthou E, Maria K, Kalliopi G, (2014) Glycemic response of a carbohydrate-protein bar with Ewe-goat whey. Nutrients 6, 2240–2250. 24926525 10.3390/nu6062240 PMC4073147

[22] Stenvers DJ, Schouten LJ, Jurgens J, (2014) Breakfast replacement with a low-glycaemic response liquid formula in patients with type 2 diabetes: a randomised clinical trial. Br J Nutr 112, 504–512. 25091284 10.1017/S0007114514001123

[23] Mottalib A, Mohd-Yusof B-N, Shehabeldin M, (2016) Impact of diabetes-specific nutritional formulas versus oatmeal on postprandial glucose, insulin, GLP-1 and postprandial lipidemia. Nutrients 8, 443. 10.3390/nu8070443 PMC4963919 27455318

[24] Devitt AA, Oliver JS, Hegazi RA, (2012) Glycemia targeted specialized nutrition (GTSN) improves postprandial glycemia and GLP-1 with similar appetitive responses compared to a healthful whole food breakfast in persons with type 2 diabetes: a randomized, controlled trial. J Diab Res Clin Met 1, 20.

[25] Jakubowicz D & Froy O (2013) Biochemical and metabolic mechanisms by which dietary whey protein may combat obesity and type 2 diabetes. J Nutr Biochem 24, 1–5. 22995389 10.1016/j.jnutbio.2012.07.008

[26] Mignone LE, Wu T, Horowitz M, (2015) Whey protein: the “whey” forward for treatment of type 2 diabetes? World J Diabetes 6, 1274–1284. 26516411 10.4239/wjd.v6.i14.1274 PMC4620107

[27] Comerford KB & Pasin G (2016) Emerging evidence for the importance of dietary protein source on glucoregulatory markers and type 2 diabetes: different effects of dairy, meat, fish, egg, and plant protein foods. Nutrients 8, 446. 10.3390/nu8080446 PMC4997361 27455320

[28] Chen Q & Reimer RA (2008) Dairy protein and leucine alter GLP-1 release and mRNA of genes involved in intestinal lipid metabolism in vitro. Nutrition 25, 340–349. 19036562 10.1016/j.nut.2008.08.012 PMC3827015

[29] Reimer RA (2006) Meat hydrolysate and essential amino acid-induced glucagon-like peptide-1 secretion, in the human NCI-H716 enteroendocrine cell line, is regulated by extracellular signal-regulated kinase 1/2 and p38 mitogen-activated protein kinases. J Endocrinol 191, 159–170. 17065399 10.1677/joe.1.06557 PMC3838362

[30] Manders RJF, Hansen D, Zorenc AHG, (2014) Protein co-ingestion strongly increases postprandial insulin secretion in type 2 diabetes patients. J Med Food 17, 758–763. 24611935 10.1089/jmf.2012.0294

[31] Almario RU, Buchan WM, Rocke DM, (2017) Glucose-lowering effect of whey protein depends upon clinical characteristics of patients with type 2 diabetes. BMJ Open Diabetes Res Care 5, e000420. 10.1136/bmjdrc-2017-000420 PMC5530249 28761664

[32] Sun L, Ranawana DV, Leow MK, (2014) Effect of chicken, fat and vegetable on glycaemia and insulinaemia to a white rice-based meal in healthy adults. Eur J Nutr 53, 1719–1726. 24817596 10.1007/s00394-014-0678-z

[33] Williams JA, Almeida JG, Martin MM, (2009) Amount and type of carbohydrate in diabetes-specific nutritional formulas affects glycemic response in patients with type 2 diabetes. Clin Nutr Suppl 4, 168.

[34] Wheeler LM & Pi-Sunyer FX (2008) Carbohydrate issues: type and amount. J Am Diet Assoc 108, 34–39. 10.1016/j.jada.2008.01.024 18358253

[35] Nuttall FQ (1993) Dietary fiber in the management of diabetes. Diabetes 42, 503–508. 8384131 10.2337/diab.42.4.503

[36] Ur-Rehman S, Mushtaq Z, Zahoor T, (2015) Xylitol: a review on bioproduction, application, health benefits, and related safety issues. Crit Rev Food Sci Nutr 55, 1514–1528. 24915309 10.1080/10408398.2012.702288

[37] Achour L, Méance S & Briend A (2001) Comparison of gastric emptying of a solid and a liquid nutritional rehabilitation food. Eur J Clin Nutr 55, 769–772. 11528491 10.1038/sj.ejcn.1601221

