# Supplementary material for: Postprandial effects of a whey protein-based multi-ingredient nutritional drink compared with a normal breakfast on glucose, insulin, and active GLP-1 response among type 2 diabetic subjects: a crossover randomised controlled trial
Source: J Nutr Sci. 2021 Jul 12;10:e49. doi: 10.1017/jns.2021.41 (PMC8278161; doi:10.1017/jns.2021.41)
Supplement: Supplementary file 1 [file jnssup.zip › S2048679021000410sup001.docx]

**Supplementary Table**

**Table S1** Baseline and postprandial plasma glucose concentrations after consumed test diets

| **Time (min)** | **Plasma glucose (mg/dL)** | |
| --- | --- | --- |
|  | **Boiled white rice with chicken; BC** | **Whey protein-based multi-ingredient nutritional drink; WD** |
| 0 (baseline) | 133 ± 6^a^ | 133 ± 7^a,d^ |
| 30 | 205 ± 8^b^ | 152 ± 7^b,c^ |
| 60 | 227 ± 10^c^ | 161 ± 8^c^ |
| 90 | 204 ± 9^b^ | 158 ± 9^c^ |
| 120 | 176 ± 9^d^ | 150 ± 9^a,b,c^ |
| 180 | 134 ± 10^a^ | 130 ± 7^a,b^ |
| 240 | 112 ± 10^a^ | 113 ± 6^d^ |

Values were mean ±SD; n=15. Mean values at each time of measurements (same column) with different superscript letters are significantly different (*P* <0.05), determined using repeated measures ANOVA and Bonferroni’s correction for post-hoc comparisons.

**Table S2** Baseline and postprandial serum insulin concentrations after consumed test diets

| **Time (min)** | **Serum insulin** **(μIU/mL)** | |
| --- | --- | --- |
|  | **Boiled white rice with chicken; BC** | **Whey protein-based multi-ingredient nutritional drink; WD** |
| 0 (baseline) | 16.7 ± 3.5^a^ | 16.7 ± 3.4^a^ |
| 30 | 42.1 ± 8.6^b^ | 39.6 ± 6.1^b,c^ |
| 60 | 65.5 ± 10.6^c^ | 47.4 ± 8.0^a,b,c^ |
| 90 | 53.6 ± 7.6^b,c^ | 51.7 ± 9.6^c^ |
| 120 | 54.2 ± 13.6^b,c^ | 32.0 ± 5.9^a,b,c^ |
| 180 | 34.0 ± 6.2^b^ | 34.3 ± 5.7^a,b,c^ |
| 240 | 31.0 ± 7.2^a,b^ | 19.1 ± 3.4^a,b^ |

Values were mean ±SD; n=15. Mean values at each time of measurements (same column) with different superscript letters are significantly different (*P* <0.05), determined using repeated measures ANOVA and Bonferroni’s correction for post-hoc comparisons.

**Table S3** Baseline and postprandial plasma active GLP-1 concentrations after consumed test diets

| **Time (min)** | **Plasma active GLP-1 (pM/mL)** | |
| --- | --- | --- |
|  | **Boiled white rice with chicken; BC** | **Whey protein-based multi-ingredient nutritional drink; WD** |
| 0 (baseline) | 3.9 ± 0.5^a^ | 5.1 ± 1.6^a^ |
| 30 | 9.4 ± 1.1^b^ | 16.7 ± 3.3^b,c^ |
| 60 | 7.6 ± 1.2^b^ | 17.5 ± 4.0^c^ |
| 90 | 8.4 ± 1.3^b^ | 18.7 ± 3.8^c^ |
| 120 | 7.1 ± 1.0^b^ | 16.0 ± 3.6^b,c^ |
| 180 | 8.0 ± 1.1^b^ | 13.2 ± 3.5^b^ |
| 240 | 7.8 ± 1.4^b^ | 9.0 ± 2.9^a^ |

Values were mean ±SD; n=15. Mean values at each time of measurements (same column) with different superscript letters are significantly different (*P* <0.05), determined using repeated measures ANOVA and Bonferroni’s correction for post-hoc comparisons.
