# Supplementary figures and images for: Postprandial effects of a whey protein-based multi-ingredient nutritional drink compared with a normal breakfast on glucose, insulin, and active GLP-1 response among type 2 diabetic subjects: a crossover randomised controlled trial
Source: J Nutr Sci. 2021 Jul 12;10:e49. doi: 10.1017/jns.2021.41 (PMC8278161; doi:10.1017/jns.2021.41)

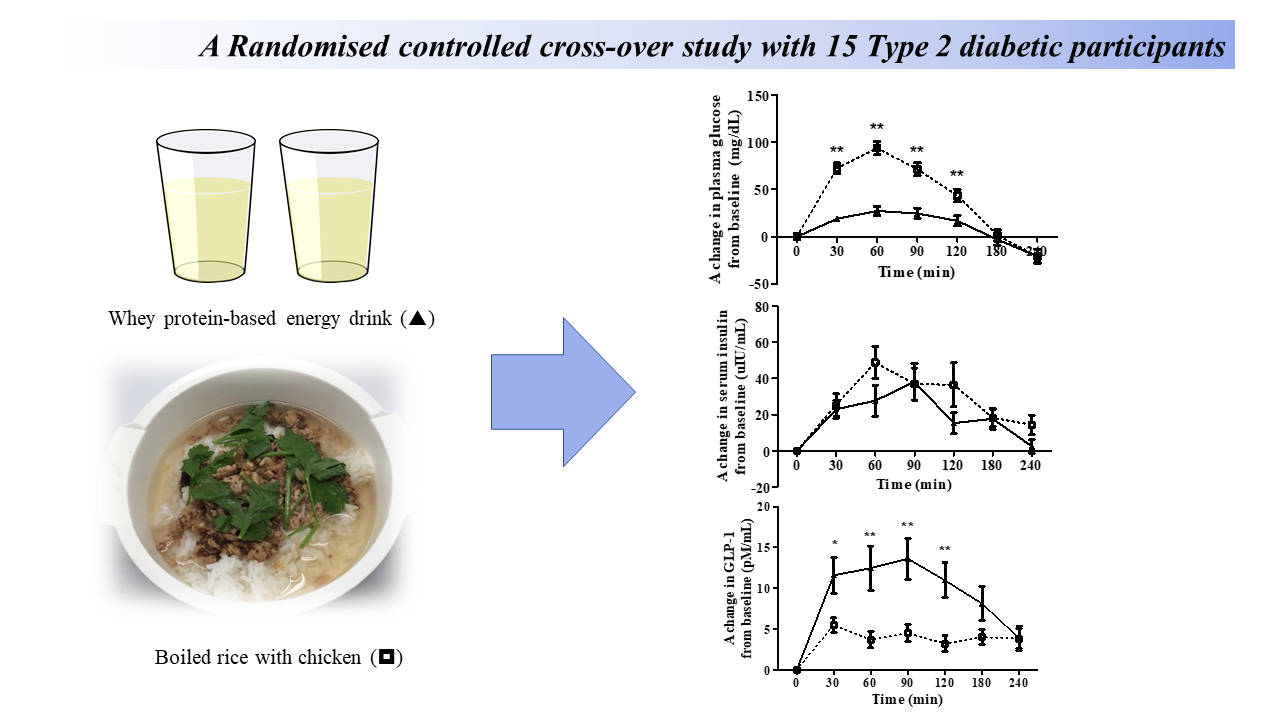

Supplement: Supplementary file 1 [file jnssup.zip › S2048679021000410sup002.tif]
